# The Neural Correlates of Abstract and Concrete Words: Evidence from Brain-Damaged Patients

**DOI:** 10.3390/brainsci3031229

**Published:** 2013-08-07

**Authors:** Costanza Papagno, Giorgia Martello, Giulia Mattavelli

**Affiliations:** Department of Psychology, University of Milano-Bicocca, Piazza dell’Ateneo Nuovo 1, Building U6, Milan 20126, Italy; E-Mails: g.martello3@campus.unimib.it (G.M.); g.mattavelli2@campus.unimib.it (G.M.)

**Keywords:** concreteness, temporal lobe, insula, associative *vs.* categorical architecture

## Abstract

Neuropsychological and activation studies on the neural correlates of abstract and concrete words have produced contrasting results. The present study explores the anatomical substrates of abstract/concrete words in 22 brain-damaged patients with a single vascular lesion either in the right or left hemisphere. One hundred and twenty (60 concrete and 60 abstract) noun triplets were used for a semantic similarity judgment task. We found a significant interaction in word type × group since left temporal brain-damaged patients performed significantly better with concrete than abstract words. Lesion mapping of patients with predominant temporal damage showed that the left superior and middle temporal gyri and the insula were the areas of major overlapping, while the anterior portion of the left temporal lobe was generally spared. Errors on abstract words mainly concerned (although at a non-significant level) semantically associate targets, while in the case of concrete words, coordinate targets were significantly more impaired than associate ones. Our results suggest that the left superior and middle temporal gyri and the insula are crucial regions in processing abstract words. They also confirm the hypothesis of a semantic similarity *vs.* associative organization of concrete and abstract concepts.

## 1. Introduction

The superior cognitive processing of concrete as compared to abstract nouns has been demonstrated in a number of psycholinguistic studies (for a review see [1]), and is the rule in aphasia (e.g., [2]). Yet, neuropsychological patients with a reversal of concreteness effect have been reported (e.g., [3,4,5]). Two main models have been proposed to explain the concreteness effect. The dual-coding theory claims that the processing of abstract nouns relies on verbal code representations of the left cerebral hemisphere only, whereas concrete nouns additionally access a second image-based processing system in the right hemisphere [1]. An alternative model, the context availability theory [6], argues that the faster recognition of concrete *vs.* abstract nouns results from a larger contextual support of concrete words and not from a distinct non verbal system; this theory does not explicitly rule out a right hemisphere involvement, but attributes the concreteness effect purely to the access of more verbal information, which implies a predominantly left-hemisphere-based processing system. These theories assume that there is a quantitative distinction between concrete and abstract concepts, but they cannot explain the presence of brain-damaged patients with a reversal of concreteness effect, *i.e.*, a superiority of abstract concepts with respect to concrete concepts. So far, several single cases have been reported in the literature with poorer concrete than abstract concept knowledge [3,4,5,7,8,9,10,11,12] (see Table 1). The data from these patients support the view that concrete and abstract words are represented in a different qualitative, as well as quantitative, way in the brain. These patients show either a unilateral left temporal lesion [7] or bilateral lesion [9] or atrophy [3,4,5], more pronounced on the left side. Crucially, patient DM [3] showed a hypoperfusion of the inferior temporal gyrus (ITG), particularly on the left side—the mean intensity score of blood flow in the left anterior ITG was approximately 10% lower than that of the right anterior ITG, the perfusion deficit being maximal at approximately 25–30 mm from the temporal tip. Patient FB [9] had an extensive bilateral lesion involving hippocampal and amygdaloid structures as well as temporal neocortex, the temporal pole and the most anterior part of the infero-temporal cortex. In Papagno *et al.* [5], VBM revealed hypodensity in the left temporal pole and medial temporal cortex. None of them had an involvement of the angular gyrus, which is usually damaged when concrete concepts are impaired (see later).

**Table 1:** Neuropsychological single cases with a reversed concreteness effect. Legend: HSE = herpes simplex encephalitis; SD = semantic dementia; CVA = cerebrovascular accident; T = temporal; P = parietal; O = occipital; ITG = inferior temporal gyrus.

Authors	Patient	Etiology	Site
Warrington 1975 [10]	AB	atrophy	bilateral
Warrington 1981 [11]	CAV	glioma	Left T-P-O
Warrington and Shallice 1984 [12]	SBY	HSE	Bilateral T
Sirigu *et al.* 1991 [9]	FB	HSE	Bilateral medial T
Breedin *et al.* 1994 [3]	DM	SD	Bilateral ITG, >left anterior
Marshall *et al.* 1996 [7]	RG	CVA	Left? (no scan)
Macoir 2008 [4]	SC	SD	Anterior T left > right
Mattioli 2008 [8]		HSE	Left T
Papagno *et al.* 2009 [5]	MC	SD	Left T pole and medial T

To explain the dissociation between abstract and concrete words, some authors [13] argue for a fundamental difference in the architecture of their representations—the primary organization of concrete concepts is categorical, whereas abstract concepts are predominantly represented by association to other items. Put differently, abstract words are assumed to be organized mainly by semantic association and concrete words mainly by semantic similarity. In this framework, a reversed concreteness effect might result from selective damage to categorical information, which would selectively affect conceptual representations of concrete words.

Neuroimaging studies have produced inconsistent results on the neural correlates of abstract and concrete words, possibly because of the different task modalities and methodologies (PET, fMRI) adopted. Differences among studies concern not only the intra-hemispheric location, but also the lateralization of the processes. Concrete word processing, relative to abstract word processing, has produced greater activation in a bilateral network of associative areas, including temporal, parietal and prefrontal cortex, while processing of abstract words produced greater activation almost exclusively in the left superior temporal and inferior frontal cortex, both when using a semantic similarity judgment task on concrete and abstract noun triads [14], or synonym judgments [15]. A bilateral activation of the angular gyrus and the dorsal prefrontal cortex was found for auditorily presented concrete words using a lexical decision paradigm [16], while there was left lateral temporal lobe activation for both types of words. A reversed pattern of activation has been observed in two PET studies [17,18]. In a lexical decision task [17], abstract word processing was associated with selective activation of the temporal pole and amygdala on the right, and of the inferior frontal cortex bilaterally, while no brain areas were more active in response to concrete words. In a semantic similarity judgment task with visually presented words [18], an area of greater activation was found on the left medial fusiform gyrus for concrete words, while a greater activation was detected on the right medial fusiform gyrus for abstract words. A lexical decision paradigm with a direct comparison between the abstract and concrete stimuli yielded a significant area of activation in the right anterior temporal cortex [19]. In this study it was also found that the right posterior temporal lobe was engaged during lexical decision for both abstract and concrete words, the statistical significance of the activation being greater for abstract words. The fusiform gyrus was activated equally by either concrete or abstract words.

Therefore, comparison between activation studies is not straightforward because of variations in tasks, methods, and material. Results can differ depending on whether lexical decision or semantic similarity judgment is required; a visual presentation is usually adopted, but in some cases, stimuli are presented auditorily. Abstract and concrete words are not always checked for imageability and abstract words present a high degree of variability within this dimension [20]. Finally, response type can have a relevant effect on results, as demonstrated in a study [21] showing a significant interaction between response type and the brain regional activation during semantic memory tasks.

A recent meta-analysis on activation studies [22] indicates that abstract concepts elicit greater activity in the inferior frontal gyrus (IFG) and middle temporal gyrus (MTG) as compared to concrete concepts, while concrete concepts elicit greater activation in the posterior cingulate, precuneus, angular gyrus, fusiform gyrus, and parahippocampal gyrus compared to abstract concepts.

There are also a few TMS studies on this topic. Using a lexical decision paradigm, an interference on accuracy for abstract words was found when repetitive (r)TMS was applied over the left temporal site, while for concrete words accuracy decreased when rTMS was applied over the right temporal site [23]. Accuracy for abstract words, but not for concrete ones, decreased after IFG stimulation. In a different study using offline rTMS with a synonym judgment task [24], disruption of the left or right temporal pole considerably slowed the time required to make semantic decisions, both with abstract and concrete concepts, but did not affect accuracy. However, the authors consider imageability synonymous with concreteness, which is not the case (see for example [25], and more recently, [26]). Indeed, imageability is a property of a word or concept reflecting how easy it is to visually or acoustically represent it, while concreteness indicates items whose meanings are mainly acquired through perceptual experience. Although concrete material is mostly imageable, abstract words present a high degree of variability within this dimension [20] and some abstract words can be highly imageable (for example, emotional words). Moreover, stimulation cannot exclusively reach (whenever it does) the temporal pole; finally, in TMS studies only a limited number of sites can be investigated at a time. 

In the light of the above, we aimed at examining a series of brain-damaged patients with a single vascular lesion either in the left or right hemisphere, involving the temporal or the frontal lobe to further investigate the neural correlates of abstract and concrete concepts by means of a semantic similarity judgment task, including 120 (60 abstract and 60 concrete) noun triplets, randomly intermingled. The target words were semantically associated in 60 trials (30 for concrete and 30 for abstract nouns) or semantically coordinate in the remaining 60. Our hypothesis was that patients with left inferior frontal or temporal posterior damage would be impaired with abstract, but not concrete words, while patients with more anterior temporal lesions would show impairment with concrete, but not abstract words.

## 2. Results and Discussion

Three right brain-damaged patients (RBD) showed unilateral spatial neglect (USN) but none had signs of cognitive decline at the time of testing. However, we checked that they were able to read both alternative words on the screen. All RBD patients had consistent damage to the temporal lobe, minimally extending in the frontal or parietal region. Seven left brain-damaged patients (LBD) had a fluent aphasia, while three were classified as non-fluent aphasics and two showed no aphasia at the time of testing (see Table 2, Table 3 for patients’ demographical and clinical data). The mean Token Test (TT) score was 25.41 (SD 5.29, range 18–35). Eight LBD patients had a lesion involving mainly the temporal lobe, while in four the damage concerned more consistently the frontal lobe. Temporal LBD and RBD did not differ in age [*t*(16) = −0.8, *p* = 0.41] or educational level [*t*(16) = −1.6, *p* = 0.12].

Left and right temporal patients as well as controls were compared by means of an ANOVA for repeated measures on accuracy and mean RTs, with word type (two levels: abstract *vs.* concrete) as within subject factor and group (three levels: controls, LBD and RBD) as between subject factor. The four patients with a frontal lesion were examined as single cases [27]. 

**Table 2:** Demographical and clinical data of the 10 RBD and the 12 LBD included in the study. Legend: R = right; L = left; F = frontal, T = temporal, P = parietal, ins = insula; n.a. = not assessed, USN = unilateral spatial neglect. TT cut-off > 29; conventional BIT cut-off < 129; MMSE ≥ 26.

Patient	Sex	Age	Education	Side	Site	TT	BIT	MMSE	Deficits
BM	F	50	8	R	T		146/146	n.a.	L hemiparesis
SM	M	63	8	R	F-T		142/146	n.a.	L hemiparesis
TM	F	65	11	R	P-T		145/146	24/30	-
DA	M	50	8	R	F-T		117/146	25/30	USN
RC	F	59	17	R	F-T		126/146	27/30	USN
FN	M	45	8	R	F-T		111/146	28/30	USN
AA	M	55	10	R	F-T-P		146/146	n.a.	L hemiplegia
CA	M	57	13	R	F-T-P		146/146	28/30	L hemiplegia
MA	M	59	13	R	T		62/146	26/30	L hemiparesis
AR	M	57	13	R	T ins.		50/146	28/30	L hemiparesis
TF	M	67	13	L	T-P	22			Wernicke aphasia
GS	M	72	13	L	F	28			R hemiplegia
AS	F	43	13	L	T-P	35			-
CL	M	72	17	L	F-P	19			Wernicke aphasia
TA	M	76	8	L	T-P	18			Fluent aphasia
NA	M	68	13	L	T-O	26			Fluent aphasia
GG	M	63	13	L	F	29			Non fluent aphasia
FC	M	70	8	L	T	29			Fluent aphasia
ZP	M	59	17	L	T-P-O	25			Fluent aphasia
ML	F	55	13	L	T-P	26			Brocaaphasia
MM	M	70	13	L	F-P	18			Non fluent aphasia
CP	F	40	18	L	T	30			Fluent aphasia

**Table 3:** Language examination scores of the 12 LBD patients. Since patients were evaluated in different structures and therefore submitted to different standardized batteries, scores are reported in percentage of correct responses; n.a. = not available.

Patient	Naming	Repetition	Comprehension
TF	86.6%	60%	61.3%
GS	n.a.	n.a.	n.a.
AS	66.6%	100%	96%
CL	n.a.	n.a.	n.a.
TA	48.3%	72.66%	68.3%
NA	43.3%	91.3%	75%
GG	n.a.	n.a.	n.a.
FC	66.6%	n.a.	n.a.
ZP	76.6%	42.2%	95%
ML	52%	61.3%	95%
MM	55%	100%	95%
CP	82.5%	92.6%	95.8%

**Figure 1:**
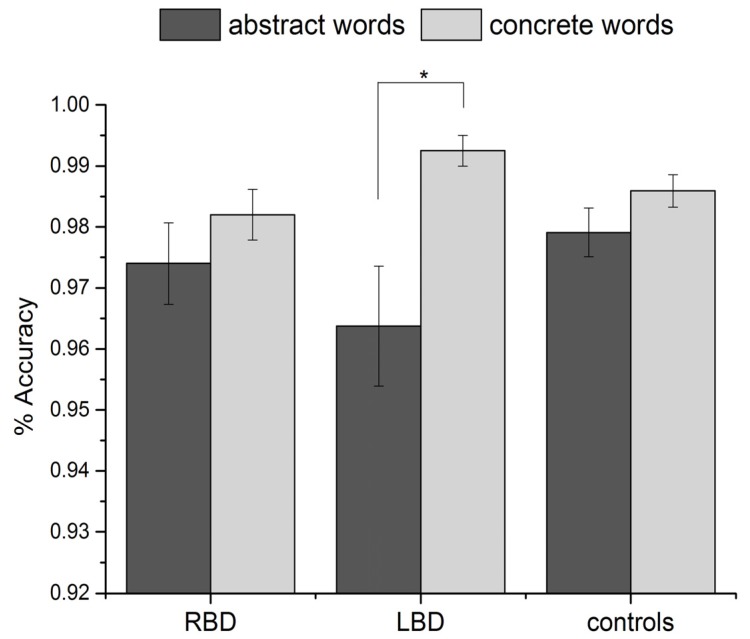
Percentage of correct responses for abstract and concrete words in RBD, temporal LBD and controls. Error bars represent standard errors of the mean.

Regarding accuracy (see Figure 1), the main effect of word type was significant [*F*(1, 37) = 16.5, *p* = 0.001, partial eta square = 0.31], with a lower number of correct responses for abstract than concrete words; the effect of group was not significant [*F*(2, 37) = 0.5, *p* = 0.6]. The interaction group × word type was significant [*F*(2, 37) = 3.5, *p* = 0.04, partial eta square = 0.16]. *Post-hoc* analyses (Tukey test) showed that left temporal patients produced significantly more errors with abstract than concrete words (*p* = 0.004). This difference was not significant in RBD patients (*p* = 0.8) or in controls (*p* = 0.6) (see Figure 1). 

**Figure 2:**
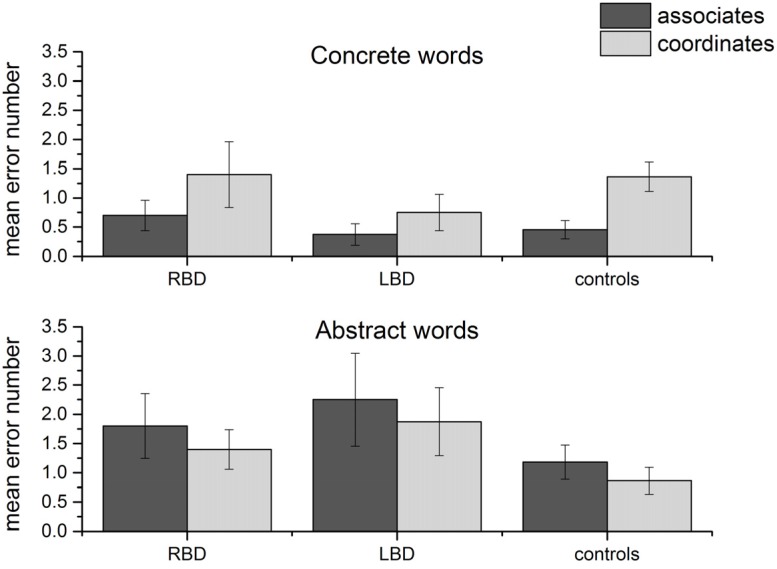
Number of errors for coordinate and associate target words in the three groups. Error bars represent standard errors of the mean.

After having verified that performance was lower for left temporal patients with abstract words, we checked whether error type differed depending on the type of item. Indeed, since the target word for each triplet could be an associate or a coordinate word, the second hypothesis was that with abstract words, errors would mainly affect associate rather than coordinate targets, while the opposite should be true for concrete words (following [13]). An ANOVA on number of errors word type (two levels: abstract and concrete) × semantic relation (two levels: associate *vs.* coordinate) × group (three levels: RBD, LBD, controls) showed a significant interaction word type × semantic relation [*F*(1, 37) = 7.89, *p* = 0.008, partial eta square = 0.18], while the three-way interaction (word type × semantic relation × group) was not significant (see Figure 2). This is not surprising since we did not expect an effect of left *vs.* right lesion *vs.* normal brain on the primary organization of concepts. *Post-hoc* analyses showed that there were significantly more errors with semantically coordinate compared to associate targets in the case of concrete words (*p* = 0.01), while the opposite was true for abstract words, although the results did not reach significance.

We then examined the four LBD patients with a predominantly frontal lesion by applying the Revised Standardized Difference Test [27] to compare each patient’s discrepancy in performance with abstract as compared to concrete items with the control sample (five matched controls for each patient). This test allows establishing whether or not discrepant scores on two subtests for a patient can be taken as a reliable dissociation. Only one patient was significantly impaired with abstract words, while for concrete words the analysis only approached significance [*t*(4) = −2.74, *p* = 0.02 and *t*(4) = −1.92, *p* = 0.06, respectively]. Crucially, the lesion included the superior part of the temporal pole. Two patients did not differ from controls both in concrete and abstract words, while the remaining one was severely impaired in both concrete and abstract words as compared to controls. His lesion, however, was the largest, involving also the parietal and the superior part of the temporal lobe. 

As expected, accuracy correlated with severity of aphasia, measured as TT score (*r* = 0.67, *p* < 0.05).

We also analysed RTs (see Table 4). RTs were excluded from the analysis when the subjects responded incorrectly. 

**Table 4:** Raw RT values for abstract and concrete words in the three groups of participants. SD are reported in brackets.

	RBD	LBD	Controls
Abstract	5006.55 (1729.9)	5338.23 (4439.2)	2453.6 (531.2)
Concrete	4298.42 (1439.8)	4649.28 (3535.1)	2238.9 (426)

There was a significant effect of word type [*F*(1, 37) = 20.9, *p* < 0.001], being all participants slower with abstract than concrete items; the effect of group was significant [*F*(2, 37) = 8.2, *p* = 0.001], but the interaction was not significant [*F*(2, 37) = 2.5, *p* = 0.093]. Pairwise comparisons (Bonferroni correction) showed that controls were faster than RBD (*p* = 0.01) and LBD (*p* = 0.006). The results did not change when we applied outlier removal procedures, such as excluding RTs > 5000 (word type *p* = 0.0001 participants being slower with abstract items, group *p* = 0.0001, controls being faster than brain-damaged patients, word type × group, *p* = 0.021, again participants being faster with concrete items).

## 3. Lesion Mapping

**Figure 3:**
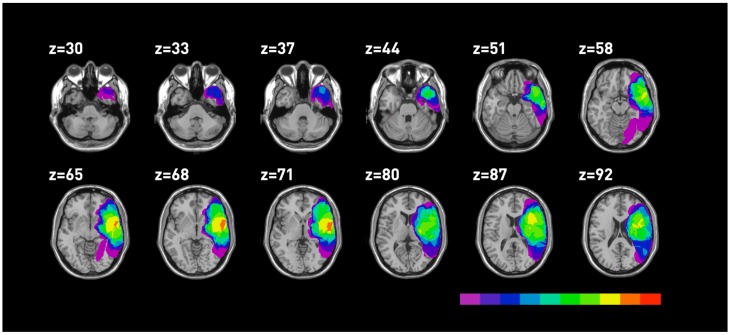
Mapping of the areas involved in RBD. Different colours correspond to the degree of lesion overlapping, with red indicating the region involved in all patients.

**Figure 4:**
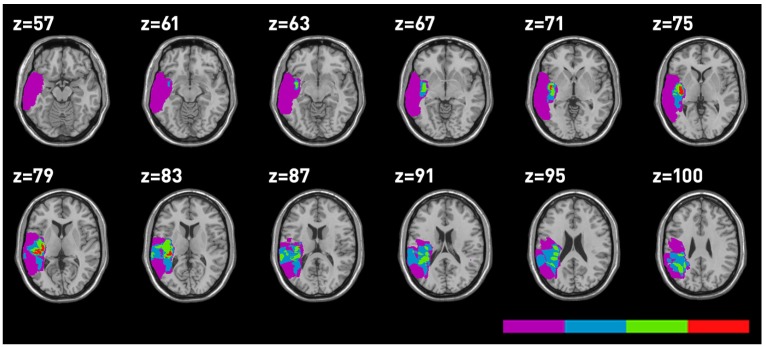
Mapping of the areas involved in temporal LBD.

Lesions were mapped using MRIcro on 17 MRI performed at the time of testing. Five MRI were unavailable. The anatomical sites, which proved to be involved in the lesion for all patients are reported in Table 5 and the lesion mappings are reported in Figure 3, Figure 4. The superior temporal gyrus (STG), the MTG and the insula were the sites of major overlapping in temporal LBD as well as RBD patients.

**Table 5:** Number of voxels involved in lesions in LBD and RBD patients. Areas of major overlapping are reported in bold.

Areas involved	Total	
	Left	Right
Precentral_	26.33	44.03
Frontal_Sup_	26.22	3.77
Frontal_Sup_Orb_	7.60	0.98
Frontal_Mid_	37.62	46.31
Frontal_Mid_Orb_	6.92	9.45
Frontal_Inf_Oper_	13.56	57.19
Frontal_Inf_Tri_	27.01	77.93
Frontal_Inf_Orb	12.41	58.77
Rolandic_Oper_	21.43	68.35
Supp_Motor_Area	8.49	
Olfactory_	2.18	0.96
Frontal_Sup_Medial	21.79	94.31
Frontal_Mid_Orb	5.72	0.57
Rectus	6.64	0.35
Insula	**39.23**	94.31
Cingulum_Ant	10.40	-
Cingulum_Mid	2.04	0.57
Hippocampus	0.43	5.31
ParaHippocampal	0.48	5.47
Amygdala	0.48	4.76
Occipital_Sup	0.13	0.22
Occipital_Mid	4.82	3.71
Occipital_Inf	0.01	2.56
Fusiform	-	6.23
Postcentral	26.21	45.52
Parietal_Sup	0.16	4.05
Parietal_Inf	7.60	17.66
SupraMarginal	14.33	35.39
Angulargyrus	10.20	21.09
Precuneus	0.04	0.49
Paracentral_Lobule	0.12	0.04
Caudate	7.78	14.97
Putamen_	12.71	42.02
Pallidum_	1.72	8.25
Thalamus_	0.37	7.47
Heschl_	7.69	11.80
Temporal_Sup	**44.7**	137.64
Temporal_Pole_Sup	9.71	64.86
Temporal_Mid	**40.69**	126.51
Temporal_Pole_Mid	1.24	33.57
Temporal_Inf	8.29	39.78

In general, patients were not really impaired and the level of accuracy was high overall. Yet, some interesting results emerged. We will first consider the negative data, namely an absence of impairment for concrete words in left temporal patients and the lack of deficits after a right temporal lesion. Both groups of patients did not significantly differ from controls in the case of concrete words, although it is possible that since controls responded significantly faster than both groups of patients, they produced a few more errors. In fact, when we performed an ANOVA on RT/accuracy, we found a significant effect of word type [*F*(1, 37) = 26.83, *p* < 0.001, partial eta square = 0.42], a significant effect of group [*F*(2, 37) = 8.29, *p* = 0.001, partial eta square = 0.31], since controls performed significantly better than RBD (*p* = 0.009) and LBD (*p* = 0.006). The interaction word type × group was also significant [*F*(2, 37) = 8.29, *p* = 0.022, partial eta square = 0.31]: while controls did not differ in performance with abstract and concrete words (*p* = 0.7), both RBD (*p* = 0.009) and LBD (*p* = 0.01) did, with a significantly better performance with concrete items. Therefore, if concrete words were bilaterally represented, as suggested by some activation studies (for example [28]), we should have found the opposite pattern. In addition, both the presence of LBD patients with a reversed concreteness effect and the absence of a bilateral activation in some neuroimaging studies (see for example [29,30]) challenge the hypothesis of a bilateral representation of concrete terms. Concerning the role of the left temporal lobe in processing concrete words, its anterior part was never damaged, except in one case. Crucially, a single case analysis performed on this patient’s accuracy [27] showed a classical dissociation since the patient’s discrepancy was significantly different from the control sample [*t*(4) = 5.49, *p* = 0.005, for concrete words *t*(4) = 0.000, *p* = 0.5, for abstract words *t*(4) 0–6.16, *p* = 0.002]. Of course, a single case cannot be conclusive, but it is in line with the data on SD patients and with a recent study conducted on patients with selective anterior temporal lobe resection [31]. In addition, in our series, patients did not suffer significant damage to the angular gyrus, which may explain why concrete word processing was not impaired.

We will now discuss the significant impairment of abstract words when the left STG and MTG and the insula were damaged. The involvement of the insula is not completely new, since it was found when comparing abstract words to pseudo-words [16]. One could argue that processing emotional words especially with a negative valence [32] involves the insula. Although we carefully selected abstract words, in order to avoid those with an emotional content, some of them (e.g., jealousy, goodness) did convey either a positive or negative valence, possibly explaining our results. In order to investigate this issue we asked a different group of 30 healthy subjects (15 male, mean age 36.3 years, SD 13.8, range 24–67, mean educational level 17.5 years, SD 1.6, range 11–18) to rate the emotional valence of each stimulus word from −9 (totally negative) to 9 (totally positive) considering the range between −3 and 3 as neutral. The effect of valence was then studied by means of a repeated measure ANOVA with valence (negative, positive, neutral) and lesion side (RBD, LBD) as independent factors. We found a significant effect of valence [*F*(2, 32) = 4.34, *p* = 0.021], accuracy being lower for negative than positive items (*p* = 0.012), while there was no significant effect of group [*F*(1, 16) = 0.002, *p* = 0.96]. The interaction was not significant [*F*(2, 32) = 0.069, *p* = 0.93]. Therefore, although emotional valence affects performance, this alone cannot explain the lower performance of LBD with abstract words, since the valence effect was evident in both groups of patients.

Regarding the involvement of the MTG, this is in line with the meta-analysis reported above [22] that indicates the IFG and the MTG as the sites where abstract concepts elicited greater activity. In addition, the STG showed a greater activation for abstract words in some previous studies (e.g., [28,33]) and more recently [34], it has been found that abstract sentences activate superior temporal and inferior frontal regions. 

## 4. Experimental Section

### 4.1. Materials and Method

#### 4.1.1. Participants

Twenty-two (seven females and 15 males) brain-damaged Italian speakers (mean age 59.77, SD 10, range 40–76; mean educational level 12.54, SD 3.23, range 8–18; interval stroke-experimental session 1–12 months, mean 5.76, SD 3.75) took part in the experiment. Ten patients had a right temporal lesion, eight a left (mainly) temporal and four a (predominant) frontal/fronto-parietal damage. Twenty-two healthy controls (mean age 59, SD 9.45, range 40–74; mean educational level 12.5, SD 3.17, range 8–18), matched for age, education and sex to the patients, also performed the experiment. All participants were right-handed with a mean score on the Edinburgh Handedness Inventory (EHI) of +98% [35]. All RBD patients (four females, mean age 55.9, range 45–65, SD 6.17; mean education 11.1, range 8–17, SD 3.28; mean interval post-stroke in months 5.2, SD 3.8) were submitted to the Mini Mental State Examination to exclude cognitive decline, and to the conventional subtests of the Behavioural Inattention Test (BIT, [36]) to assess USN. LBD patients (three females, mean age 63, SD 11.64, range 40–76; mean education 13.75, SD 2.77, range 8–18; mean interval post-stroke in months 6.2, SD 3.74) were submitted to a standard language examination and to the TT [37]. All patients had completed at least a three-month treatment for aphasia. Only patients with a TT > 15 were included in the study to make sure that instructions could be comprehended. An additional LBD patient, despite a TT of 22, had to be excluded since he was unable to complete the experimental task. All patients signed an informed consent prior to starting the experiment.

#### 4.1.2. Material

A semantic similarity judgment task was used (PACCAS, Parole Astratte e Concrete Coordinate e Associate Semanticamente; Abstract and concrete words, semantically coordinate and associate) [38]. The test included 120 (60 abstract and 60 concrete) noun triplets, randomly intermingled. Six additional stimuli were used for a training phase. The target words were semantically associated in 60 trials (30 for concrete and 30 for abstract nouns) or semantically coordinated in the remaining 60. Therefore, the same concrete/abstract word appeared twice, once with the target word being an associate and the second time a semantic coordinate. Abstract and concrete words were matched for frequency (COLFIS, [39]). The mean frequency of the stimuli was 43.5 for concrete and 47.13 for abstract words [*t*(58) = −0.37, *p* = 0.7]. The mean frequency of coordinate targets was 32.86 for concrete and 37.26 for abstract words [*t*(58) = −0.53, *p* = 0.59]. Finally, the mean frequency for semantic associate targets was 43.06 for concrete and 47.63 for abstract words [*t*(58) = −0.4, *p* = 0.69]. 

The rate of abstractness/concreteness and the conceptual proximity of pairs of words within the same triplet were checked, using a Likert scale with 30 healthy participants who did not take part in the experiment. Only concrete words with a score >4 and abstract words with a score <3 were selected. The mean rating was 4.70 (SD 0.17, range 4.23–4.98) for concrete items and 2.15 (SD 0.3, range 1.58–2.98) for abstract items. The difference was significant (*t* = −81.54, *p* = 0.001). Thirty additional healthy participants were tested to verify whether RTs were comparable for abstract and concrete items. Mean RTs for concrete coordinates were 2034 ms, while for abstract coordinates were 2066 ms. Mean RTs for concrete associate were 1819 ms, while for abstract coordinates they were 2253 ms. Similarly, percentage of errors was 2.8 for both concrete and abstract coordinate, while it was 5.5 and 3.8 for concrete and abstract associate, respectively. Differences were not significant.

### 4.2. Procedure

The test was presented by means of a computer using the software Access. Response times (RTs) and accuracy were recorded. Before starting the experiment, subjects completed a block of six practice trials (three with abstract and three with concrete stimuli). During the practice block, the participant was informed whether his/her response was correct or not. Then, in the experimental phase, no further feedback was provided.

**Figure 5:**
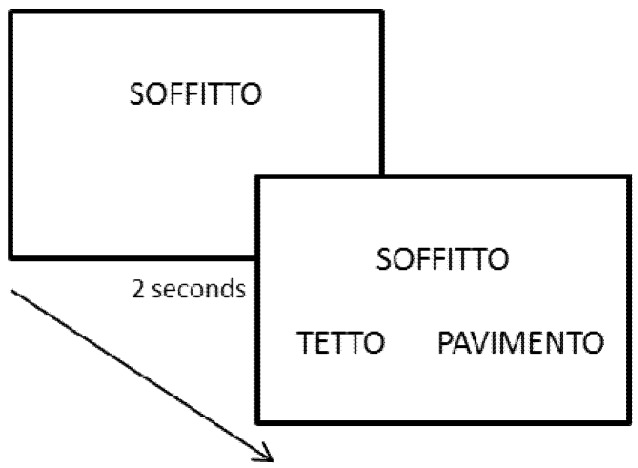
Example of the experimental task. A first word appeared centred in the upper part of the screen, followed after 2 s by the two alternative words (Soffitto = ceiling, pavimento = floor, tetto = roof).

Participants were seated in a lit room at a distance of approximately 70 cm from the computer screen. They were presented with one abstract or concrete word in a central position at the top of the screen; after 2 s, two additional words appeared at the bottom, one on the left and one on the right side (see Figure 5). The position of the correct item was randomized. The subject was required to press one of two buttons (with either hand) on the keyboard to select the word more related to the first word that had appeared. Each triplet remained on the screen until the subject responded. After each triplet, the space bar was pressed to show the next word. The total time to complete the task was approximately 20 min.

## 5. Conclusions

In order to study the neural correlates of abstract/concrete words, we investigated 10 RBD and 12 LBD patients. Only one patient had a lesion involving the IFG and, according to the previous literature, he was impaired in processing abstract words, as were patients with temporal lesions involving the left STG and MTG, as well as the insula. This last structure probably processes the emotional valence that abstract words more or less carry. More anterior-inferior parts of the left temporal lobe were spared, as it is often the case with vascular patients, and according to the literature reported in the introduction, our patients were not selectively impaired in concrete as compared to abstract words, with the exception of one case, which showed the typical damage. We are aware of the limits of our study, though: first of all, vascular lesions were not confined to the temporal or frontal lobe, and we considered where damage was prevalent; the number of patients was limited by the criteria of inclusion; not all MRI were available; and also, the experimental task was not extremely sensitive, so that the number of errors was low. Alternatively, patients produced only few errors since they were selected on the basis of their lesion and not because they presented with lexical-semantic deficits. However, one can also speculate that if the test is not difficult enough, an error becomes particularly relevant, since it indicates a real deficit. However, our results complement and support activation studies [22] and are specular to those found in SD patients where atrophy predominantly affects the anterior-inferior temporal area [3,4,5]. Moreover, our data and the existing literature suggest that the representations of abstract concepts are carried in a more distributed fashion; possibly more generally in the prefrontal cortex (see [40]). Finally, our data support the hypothesis that concrete words are organized according to a semantic similarity principle, while abstract words are predominantly organized according to a primary principle based on association, as also recently reported in healthy participants [41]. However, since our patients produced only a relatively small number of errors, this assumption still needs additional evidence. 
